# Leveraging Zebrafish to Study Retinal Degenerations

**DOI:** 10.3389/fcell.2018.00110

**Published:** 2018-09-19

**Authors:** Juan M. Angueyra, Katie S. Kindt

**Affiliations:** ^1^Retinal Neurophysiology Section, National Eye Institute, National Institutes of Health, Bethesda, MD, United States; ^2^Section on Sensory Cell Development and Function, National Institute on Deafness and Other Communication Disorders, National Institutes of Health, Bethesda, MD, United States

**Keywords:** zebrafish, retinal degeneration, regeneration, photoreceptor cells, Müller glia, developmental biology, rewiring, retinal circuitry and visual pathways

## Abstract

Retinal degenerations are a heterogeneous group of diseases characterized by death of photoreceptors and progressive loss of vision. Retinal degenerations are a major cause of blindness in developed countries (Bourne et al., 2017; De Bode, 2017) and currently have no cure. In this review, we will briefly review the latest advances in therapies for retinal degenerations, highlighting the current barriers to study and develop therapies that promote photoreceptor regeneration in mammals. In light of these barriers, we present zebrafish as a powerful model to study photoreceptor regeneration and their integration into retinal circuits after regeneration. We outline why zebrafish is well suited for these analyses and summarize the powerful tools available in zebrafish that could be used to further uncover the mechanisms underlying photoreceptor regeneration and rewiring. In particular, we highlight that it is critical to understand how rewiring occurs after regeneration and how it differs from development. Insights derived from photoreceptor regeneration and rewiring in zebrafish may provide leverage to develop therapeutic targets to treat retinal degenerations.

## Retina and Photoreceptors

**FIGURE 1:**
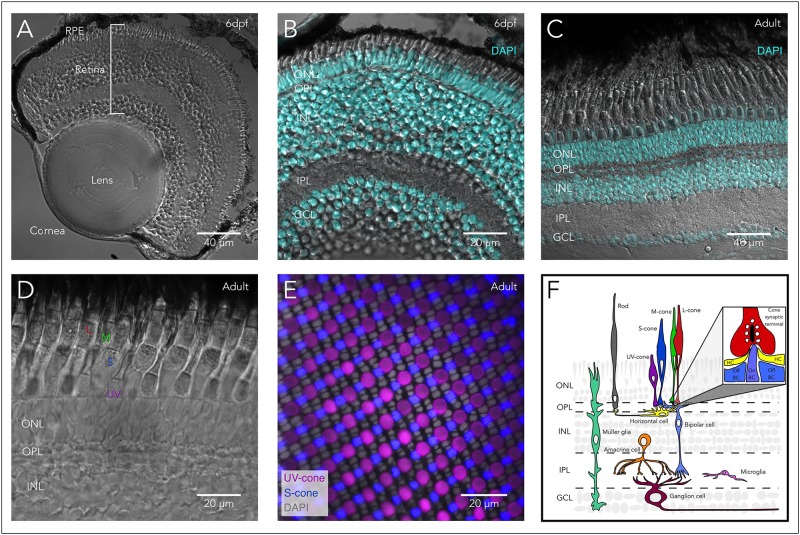
Structure of the zebrafish eye and retina. **(A)** Anatomy of the zebrafish eye: DIC image of a cryosection from a 6 days post-fertilization (6 dpf) larval eye highlighting the main structures of the vertebrate eye including cornea, lens, retina, and retinal pigment epithelium (RPE). **(B)** The larval retina is organized into highly structured layers: Overlay of a DIC image and the fluorescent nuclear marker DAPI of a cryosection from a 6 dpf larva showing the different retinal layers, including the outer nuclear layer (ONL) which contains the cell bodies of photoreceptors (rods and cones). Photoreceptors make synapses in the outer plexiform layer (OPL) with bipolar and horizontal cells. The inner nuclear layer (INL) contains the cell bodies of horizontal, bipolar and amacrine cells, while the ganglion cell layer (GCL) contains the cell bodies of retinal ganglion cells (RGC). Bipolar cells provide excitatory synaptic input to RGC in the inner plexiform layer (IPL), while amacrine cells modulate this input both pre- and post-synaptically. **(C)** The adult retina retains the same layered structure: Overlay of a DIC image and DAPI of a cryosection from an adult zebrafish. **(D)** The zebrafish retina contains 4 subtypes of cones: DIC image of a cryosection from an adult zebrafish showing the short-single or ultraviolet-wavelength sensitive cones (UV-cones), the long-single or short-wavelength sensitive cones (S-cones), and the double cones which correspond to the middle- and long-wavelength sensitive cones (M- and L-cones). **(E)** Mosaic arrangement of zebrafish cone photoreceptors: Confocal image of a whole-mounted retina of a double-reporter transgenic lines to identify UV-cones [*Tg(sws1:GFP)^kj9^*, magenta] and S-cones [*tg(sws2:mCherry)^ua3011^*, blue] overlayed with DAPI (gray), allowing the identification of the nuclei of M- and L-cones between the rows of UV- and S-cones. **(F)** Diagram of the vertebrate retina and the retinal cells. Inset highlights the synapse between cones and horizontal and bipolar cells, where the cone synaptic terminal contains synaptic vesicles (white) attached to the synaptic ribbon (black). In close apposition to the ribbon, the dendrites on On-bipolar cells (On-BCs) invaginate into the synaptic terminal and are flanked by two horizontal cell (HC) processes. Off-bipolar cells make more basal contacts in close proximity but not apposed to the synaptic ribbon.

Similar to many organs, the eye is structurally well-conserved between zebrafish and mammals. For example, the eyes of zebrafish have the same gross structure as human and other mammalian eyes, and contain a cornea, lens, vitreous, retina, pigment epithelium, choroid and sclera (**Figure 1A**). Furthermore, the development of the eye during embryogenesis is also conserved, and complimentary work in zebrafish, mice and other species has helped to delineate the key developmental events in eye morphogenesis across vertebrates (Bibliowicz et al., 2011; Stenkamp, 2015).

Within the eye, the retina is of particular interest because it is the site of sensory detection and damage to the retina results in vision loss. The vertebrate retina is a highly structured neuronal tissue that lines the back of the eye. It is responsible for both the detection and processing of visual information, before it is relayed to higher-order visual centers. To achieve this, the retina is equipped with a variety of neurons that are arranged into three nuclear layers and project into two synaptic layers (**Figures 1B,C**). Within these layers, retinal neurons assemble into multiple, distinct circuits that encode different aspects of the visual information (Gollisch and Meister, 2010). The encoding of visual information starts when light is detected by the *rod* and *cone photoreceptors*. The highly sensitive rods are mainly used during dim-lighting conditions, while the more adaptable but less sensitive cones function from dawn until dusk. The retina of the nocturnal rodents commonly used in research like mice and rats is rod dominated (97% rods and 3% cones) (Carter-Dawson Louvenia and Lavail Matthew, 1979), as is the peripheral human retina. In contrast, zebrafish have a cone-dominated retina (∼40% rods and ∼60% cones) (Fadool, 2003), similar to the central human retina, which provides high-acuity vision, and is essential for most day-to-day visual tasks. Therefore, the zebrafish retina is uniquely positioned to understand the molecular mechanisms relevant to development and regeneration of the photoreceptors that are most relevant for human vision.

The zebrafish retina contains four different cone photoreceptor subtypes (UV-, S-, M-, and L-cones). Each subtype is defined by specific opsin expression that confers a particular wavelength-sensitivity, and morphology (short or long, and single or paired with another cone type) (**Figures 1D,E**). UV-cones express *sws1*, an opsin with peak sensitivity (λ_max_) in the ultraviolet range (λ_max_ = 354 nm), and are short-single cones morphologically. S-cones express *sws2*, with peak sensitivity at short wavelengths (λ_max_ = 416 nm), and are long-single cones morphologically. M-cones express opsins of the Rh2 class, which have undergone tandem quadruplications (Rh2-1 to Rh2-4), with peak sensitivities at mid wavelengths (λ_max_ = 467 nm, 476 nm, 488 nm, and 505 nm respectively). L-cones express one of two tandemly duplicated opsin genes from the *lws* class, with peak sensitivities at longer wavelengths (λ_max_ = 548 nm and 558 nm). M- and L-cones are morphologically arranged as a double cone, where the L-cone is the long (or principal) member of the pair and the M-cone is the short (or accessory) member (Raymond et al., 1996; Vihtelic et al., 1999; Chinen et al., 2003).

In both rod and cone photoreceptors, light triggers the activation of opsins followed by the rest of the phototransduction cascade. In zebrafish and in mammals, this cascade ultimately leads to changes in photoreceptor membrane potential, and to modulation of neurotransmitter release in the synaptic terminal. Visual information is then directly transmitted from photoreceptors to several subtypes of *horizontal* (inhibitory interneurons that locally modulate photoreceptor synaptic output) and *bipolar cells* (glutamatergic neurons that transmit light signals into the next processing layer). Information is further processed in the next synaptic layer, where bipolar cells (BC) provide excitation to *ganglion cells* (glutamatergic spiking neurons), while *amacrine cells* (local interneurons) provide modulation pre- and/or post-synaptically. The axons of the retinal ganglion cells (or RGCs) form the optic nerve, and relay the pre-processed visual information to central targets in the brain. Additionally, the retina contains *microglia* (resident immune cells located primarily in the synaptic layers) and two types of true glial cells: *Müller cells* (a type of radial glia) and *astrocytes* (associated with axons of RGCs) (**Figure 1F**). The photoreceptors also closely associate with the *retinal pigment epithelium* (RPE), which provides structural, trophic and metabolic support and is directly involved in the recycling of opsins.

## Retinal Degenerations

Within the retina, rods and cones are particularly vulnerable to metabolic, genetic or environmental insults; phototransduction and synaptic signaling are demanding processes that require high metabolic rates. Retinal degenerations (RD) are disorders characterized by photoreceptor death and can affect both rods and cones, or each photoreceptor type individually. RD have multiple causes that can be broadly divided based on whether the cause is primary or secondary. In primary RD, photoreceptors are directly affected, for example due to mutations that affect phototransduction proteins. In secondary RD, other cells types, for example the RPE or a single type of photoreceptor degenerates; this can lead to secondary degeneration of other photoreceptors. The main forms of RD in humans are *Age-related Macular Degeneration* (AMD), *Retinitis Pigmentosa* (RP), and *Leber Congenital Amaurosis* (LCA). AMD is a multi-factorial disease that affects the RPE. Advanced RPE dysfunction leads to secondary rod and cone loss. In industrialized countries, AMD is the leading cause of blindness (De Bode, 2017). AMD is associated with age, smoking, nutritional deficiencies, inflammation and mutations or polymorphisms in more than 30 genes, with many more genes still to uncover (Jager et al., 2008; Warwick and Lotery, 2018). RP encompasses a set of complex hereditary diseases that can be caused by a plethora of mutations mapped to more than 70 human genes (Daiger et al., 2013; Farrar et al., 2017)^1^. Most forms of RP in humans are characterized by an initial death of rods, with subsequent secondary cone death. RP presents clinically first with night-blindness, followed by decreases in peripheral visual acuity (tunnel vision) that eventually progresses toward the central areas of the retina. LCA encompasses a group of early-onset and progressive rod and cone dystrophies. Again, 25 genes have already been identified as causes of LCA, and most are expressed either in photoreceptors or in the RPE (Kumaran et al., 2017).

Early forward genetic screens in zebrafish that evaluated defects in the histology of the photoreceptor layer identified many genes that cause RD (Brockerhoff and Fadool, 2011). Most human homologs of these genes have also been shown to cause RD in humans. Subsequent screens in zebrafish assayed for defects in visual function rather than morphological defects. These screens exploited reliable visual behavioral assays in larvae, including the optokinetic response (OKR) (Brockerhoff et al., 1995) or the escape response to moving dark objects (Li and Dowling, 1997) (see below). Together these screens helped, not only to establish additional genetic models for photoreceptor degenerations, but also to identify additional and in some cases novel genes involved in RD. For example, one zebrafish mutant that lacked OKR was linked to a mutation in *pde6c*, a novel cone-specific phototransduction gene. Mutations in *pde6c* cause cone degeneration in zebrafish with secondary rod degeneration (Stearns et al., 2007). The discovery of this mutant was beneficial in two ways. First *pde6c* mutants were used to confirm the existence of rod-specific progenitors in the zebrafish retina (Morris et al., 2008). Second this mutant was used to identify *pde6c* as the causative gene in cone-photoreceptor loss of function 1 (*cpfl1*), a specific type of cone degeneration in mouse and humans (Chang et al., 2009). This is an excellent example of how zebrafish can be used to identify novel genes and pathways involved in RD. More recently, zebrafish models of RD are being leveraged in pharmacological screens, to test or find novel treatments for human RD (Moosajee et al., 2008; Ganzen et al., 2017). Overall these zebrafish genetic screens highlight the conservation of molecules underlying RD between zebrafish and mammals. Given this level of conservation, it is likely similar molecules may be required to regenerate and rewire the zebrafish and mammalian retina after RD.

## Therapies for RD

In humans, photoreceptor loss in RD is permanent (regardless of their diverse causes and speed of progression) and therefore remain largely untreatable and lead to progressive loss of vision and ultimately blindness. In early stages of RD in humans, current treatments include neuroprotective agents (Trifunovic et al., 2012) and antibody therapies in cases where the underlying mutation is well characterized (Lazic and Gabric, 2007). Unfortunately, these treatments only slow down the progression of disease and have variable outcomes (Pardue and Allen, 2018). In addition to these treatments, gene therapy has been used to improve vision in patients with LCA caused by mutations in *RPE65*, an RPE-specific protein involved in the recycling of retinoids (Cideciyan, 2010; Jacobson et al., 2012), but the improvement may not be long-lasting (Jacobson et al., 2015).

In late stages of RD in humans, when there is widespread photoreceptor loss, two distinct approaches for treatment exist. The first seeks to bypass the need for photoreceptors. This can be achieved by either making the surviving retinal bipolar or ganglion cells photosensitive using optogenetics (Busskamp et al., 2010; Yue et al., 2016) or synthetic photo-switchable compounds (Polosukhina et al., 2012; Tochitsky et al., 2017). Additionally, retinal prostheses capable of stimulating RGCs directly have been developed, attempting to encode visual information directly into these output neurons (da Cruz et al., 2016; Lewis et al., 2016). Of note, the use of retinal prostheses for blindness was approved by the European Union in 2011, and by the FDA in 2013. Use of these prostheses has led to some successful reacquisition of very basic visual functions but only for limited periods of time (Mills et al., 2017). The second and more promising approaches aims to replace the lost photoreceptors by transplantation or by stimulating regeneration. These later approaches have received special attention because they have the capability of renewing the native function of the retina, and could provide a real cure for RD. Due to this potential for complete functional recovery, and because of recent and important developments in the field, transplantation and regeneration will be a focus of this review.

## Barriers in Photoreceptor Transplantation as a Therapy for RD

Just a decade ago, the prospect of producing photoreceptors from stem cells seemed like an overly daunting task (Adler, 2008). Nevertheless, in the last few years, several laboratories have successfully developed protocols to produce eyecup-like structures from induced-pluripotent stem cells (iPSCs) in the span of weeks. Some of these eyecups are able to acquire a layered structure reminiscent of the retina, with photoreceptor-like cells that contain outer segments, express phototransduction proteins (Wahlin et al., 2017), and have some capacity for light responsiveness (Zhong et al., 2014), and vesicular release (Wahlin et al., 2017). The successful development of these eyecups opens the possibility of harvesting cells from an individual to generate iPSCs, and re-differentiate them into photoreceptors that could be then transplanted back into patients with RD. Based on these prospects, recent work in the retinal field has focused on using mice to explore photoreceptor transplantation as a therapy for RD.

Initial transplantation studies in mice attempted to introduce rod photoreceptors, with the best rates of integration (never surpassing a few percent) achieved by transplanting immature rod precursors (MacLaren et al., 2016). Follow-up studies presented equally promising examples of integration, and in some cases demonstrated functional recovery of vision (Santos-Ferreira et al., 2015; Smiley et al., 2016). However, it has recently been discovered that many of these results are due to the exchange of cytoplasmic material (including RNA and/or proteins) between donor cells and the host retina, and *not integration* of transplanted photoreceptors (Pearson et al., 2016; Santos-Ferreira et al., 2016; Singh et al., 2016; Ortin-Martinez et al., 2017). In light of this recent discovery, it will be important to carefully interpret how functional recovery of vision occurred after transplantation/cytoplasmic exchange in degenerated or degenerating retinas (Homma et al., 2013; Singh et al., 2013; Santos-Ferreira et al., 2015). Even if cytoplasmic exchange results in recovery photoreceptor function, it occurs at very low rates (a few percent of host positive cells, for transplantations of tens to hundreds of thousands of donor cells). Such low rates casts doubt on the prospect of leveraging this process as a viable therapeutic strategy, especially in advanced cases of degeneration.

Even with viable evidence for successful photoreceptor transplantation (Waldron et al., 2018), there are additional concerns for this type of therapy. For example, subretinal injection of a mass of cells, the most common transplantation method, causes inflammation and scarring, and inhibits the migration of transplanted cells (Barber et al., 2013). Additionally, it is also unclear if transplanted photoreceptors are capable of rewiring properly into the host retina. This problem is further compounded by our incomplete understanding of how photoreceptors normally wire during development. To date, only a handful of genes are known to be involved in synapse formation between photoreceptors and their postsynaptic targets (Simmons et al., 2017; Zhang et al., 2017; Miller et al., 2018; Sarria et al., 2018; Ueno et al., 2018). Despite this work, we still have little insight on the molecules that drive the initial recognition between these cells, or on the processes that promote, inhibit or refine synapse formation. Unveiling genes involved in photoreceptor synapse formation during normal development in zebrafish could provide direct therapeutic targets to promote rewiring of transplanted photoreceptors.

## Using Zebrafish to Explore Retinal Regeneration as a Therapy for RD

Cumulatively, work on transplantation therapies has highlighted that alternative therapies, such as photoreceptor regeneration, could be a promising alternative. Unfortunately, in mammals there is no regeneration in the retina after damage or RD. In contrast, the zebrafish retina has the innate capacity for regeneration. This capacity may be due to the continued growth of the zebrafish retina into adulthood, as well as the ability of the zebrafish to maintain a population of multipotent stem cells within the retina.

Larval zebrafish form a functional visual system by 4 days post fertilization (4 dpf), and are able to perform complex visual guided behaviors (like prey capture, see below) by 5 dpf (Patterson et al., 2013). This rapid onset of sensory function is critical to survival of the animal. As larvae progress into adulthood, zebrafish continue to grow in size. This growth requires organs like the eye and retina to grow as well. In the retina this growth occurs in the ciliary marginal zone (CMZ). The CMZ maintains a niche of pluripotent cells at the edge of the retina that continually adds neurons in peripheral concentric rings (Centanin et al., 2011). In addition to this continual growth, zebrafish can also regenerate their retinas after injury. In fact, robust retinal regeneration and rewiring have been demonstrated in genetic models of RD and in other models that incur retinal injury. Overall, given that zebrafish is a genetically tractable model with active retinal regeneration, it is poised to uncover the molecular processes that control retinal regeneration and rewiring.

In teleost fish, retinal regeneration after injury has a rich history. It was first reported in goldfish (Lombardo, 1968) and later in cichlids (Johns and Fernald, 1981) and trout (Julian et al., 1998). In zebrafish retinal regeneration is robust after resection (Cameron, 2000), mechanical damage (Fausett and Goldman, 2006), light damage (Bernardos et al., 2007; Thomas et al., 2012), thermal damage (Raymond et al., 2006), pharmacological damage (Fimbel et al., 2007; Sherpa et al., 2008; Nagashima et al., 2013; Tappeiner et al., 2013; Sherpa et al., 2014) and selective ablation of particular cell-types (Montgomery et al., 2010; D’Orazi et al., 2016; Hagerman et al., 2016; Yoshimatsu et al., 2016; White et al., 2017). In teleosts, regeneration can occur from cells generated in the CMZ (Raymond et al., 2006), a dedicated population of progenitors that are committed to a rod fate (Bernardos et al., 2007; Morris et al., 2008), and the Müller glia (Fausett and Goldman, 2006; Bernardos et al., 2007; Fimbel et al., 2007).

Due to its location at the edge of the retina, the CMZ is only involved in regeneration if the injury involves the peripheral retina. During regeneration, the CMZ is capable of giving rise to all retinal neurons except rod photoreceptors (Stenkamp et al., 2001; Raymond et al., 2006). Instead, rods originate from rod-specific progenitors. These progenitors were first identified in goldfish and cichlids (Johns and Fernald, 1981) and were later found in other teleost fish including trout and zebrafish (Julian et al., 1998). Rod-specific progenitors are thought to be important for maintaining the density of rods as the eye grows, and lineage tracing revealed that these rod progenitors derive from Müller cells that slowly and continuously divide in the normal retina (Otteson et al., 2001; Raymond et al., 2006; Bernardos et al., 2007; Nelson et al., 2008). During regeneration, there is an expansion in the number of photoreceptor progenitors, but these mainly derive from actively dividing Müller glia. In fact, in zebrafish the Müller glia are the primary source of regenerated neurons after injury. During regeneration they can act as multipotent stem cells, dividing and differentiating into any retinal cell type (Ramachandran et al., 2010b). In contrast to zebrafish, in humans and other mammals Müller glia do not remain multipotent and therefore cannot readily replace lost neurons in the retina. Because Müller glia are the primary source of regenerated retinal neurons and can regenerate all retinal neurons, considerable work has been dedicated to understanding the differences between the Müller glia of zebrafish and mammals.

**FIGURE 2:**
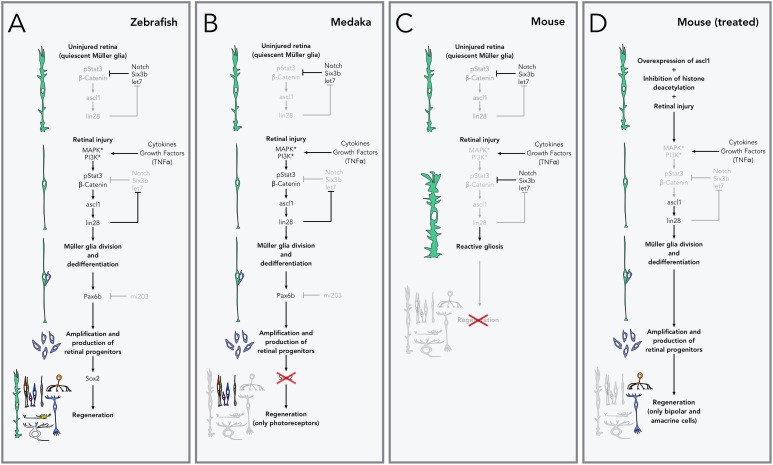
Pathways to retinal regeneration. **(A)** Zebrafish are able to completely regenerate their retina *via* Müller glia. In the uninjured retina, Müller glia is kept quiescent by inhibiting the expression of the genes that control regeneration through Notch signaling and repression by the transcription factor *Six3b* and the microRNA *let7*, amongst others. During *retinal injury*, cell death and inflammation lead to the release of cytokines and growth factors (especially TNFα), which activate receptors and kinases in the Müller glia, leading to activation of β-catenin and phosphorylation of the transcription factor *Stat3*, which in turns leads to the production of the transcription factor *ascl1* and *lin28* microRNA, and the activation of the genes that control Müller glia *division and dedifferentiation*. *lin28* also inhibits the production of *let7*, releasing the inhibition of this pathway. Expression of the transcription factor *Pax6b* (normally inhibited by the microRNA *mi203*), allows the amplification and production of retinal progenitors which are then able to redifferentiate into any retinal neurons or Müller glial cells. **(B)** In medaka fish, lack of production of *Sox2* after the production of retinal progenitors restricts their fate to photoreceptors and does not allow the production of other cell types, including new Müller glia. **(C)** In mice and other mammals, retinal injury does not lead to expression of *ascl1* and the rest of regeneration-related genes. Instead Müller glia activate the production of intermediate filaments and increase their size (*reactive gliosis*) and do not lead to the production of new retinal cells or injury repair. **(D)** Retinal regeneration can be stimulated in mice by artificially overexpressing *ascl1* and inhibiting epigenetic changes (histone deacetylation in particular). With this treatment, Müller glial cells are able to divide and produce retinal progenitors, but their fate is restricted to bipolar and amacrine cells.

A series of studies that investigated the response of zebrafish Müller glia to retinal injury, have unveiled the key transcription factors in a gene regulatory network that controls retinal repair. Shortly after injury, cytokines and growth factors activate the *beta-catenin* and *stat3* pathways (Kassen et al., 2007; Wan et al., 2014). These pathways upregulate the expression of *ascl1* (Fausett et al., 2008), a key transcription factor that (through *lin-28*) leads to the suppression of *let7* microRNA (Ramachandran et al., 2010a). In the uninjured retina, *let7* normally represses the expression of many regeneration-induced genes (including *ascl1* and *lin-28*), closing the loop of a system poised to control Müller glia response to injury (Wan and Goldman, 2016) (**Figure 2A**).

In contrast, mammalian Müller glia does not readily divide (Wan et al., 2008) and responds to retinal injury with an inflammatory response known as *reactive gliosis*, characterized by an increase in size and overproduction of intermediate filaments, and leading to distortion of the architecture of the retina without repair (Dyer and Cepko, 2000; Bringmann et al., 2009; Thomas et al., 2016) (**Figure 2C**). Significant efforts have been devoted to understanding the differences between these species, in the hope of stimulating regeneration in mammals. This work has shown that *ascl1* is not upregulated in mice after retinal injury (Karl et al., 2008), but *ascl1* overexpression in mammalian Müller cells *in vitro* is sufficient to induce production of neurons (Pollak et al., 2013). Moreover, induction of expression of *ascl1* in Müller cells *in vivo*, followed by retinal injury, induces division and production of all classes of retinal neurons, but only in young mice (Ueki et al., 2015). Recently, a successful report of regeneration in adult mice has shown that new bipolar- and amacrine-like cells derived from Müller glia can rewire into the retina. For this work, in addition to overexpression of *ascl1*, inhibition of histone deacetylation was also required (Jorstad et al., 2017). Unfortunately, no other retinal cell types are produced with this protocol (**Figure 2D**). Further insight into retinal regeneration has been gained from studying a related telost, medaka, which shows a restricted capacity for regeneration. In medaka fish, after injury, Müller glia do not readily proliferate, and new retinal progenitors commit almost exclusively to a photoreceptor fate. Comparisons in the Müller glia response to retinal injury in both medaka fish and zebrafish concluded that sustained expression of the transcription factor *sox2* in adult Müller cells is key for maintaining multipotency (Lust and Wittbrodt, 2018) (**Figure 2B**). While it is clear that important strides have been taken to attempt retinal regeneration in mammals, before regeneration can be used as a viable therapy, we need a deeper understanding on the mechanisms that maintain cells with a regenerative potential in zebrafish throughout adulthood.

## Insights from Zebrafish on Rewiring After Regeneration

Even after successful transplantation or regeneration of photoreceptors, the biggest hurdle in these RD therapies is ensuring that the new photoreceptors rewire into the appropriate retinal circuits so that they are able to restore normal visual function. Once again, zebrafish has offered a unique opportunity to study rewiring after injury and regeneration, in both larvae and adults. Cumulatively, this work has demonstrated that the extent and time course of regeneration and rewiring is determined by lesion-specific differences, in particular the extent of injury and the number of cells that need to be replaced.

Adult teleosts are able to regenerate their retinas even after extensive retinal damage. Early seminal studies in adult goldfish, and later studies in adult zebrafish, showed robust retinal regeneration and rewiring after surgical retinal extirpation (Hitchcock et al., 1992; Cameron, 2000) or pharmacologically induced death of all retinal neurons (Raymond et al., 1988; Sherpa et al., 2008). Under these lesion paradigms, all retinal cell types were regenerated. Importantly, with regards to rewiring, the retinal lamination was reestablished (Raymond et al., 1988; Sherpa et al., 2008), and synaptic connections were reformed between retinal neurons (Hitchcock and Cirenza, 1994) and between RGCs and the brain (Stuermer et al., 1985). Parallel work demonstrated that after extensive retinal damage in adult goldfish and zebrafish, visual function is also recovered (Mensinger and Powers, 1999, 2007; Lindsey and Powers, 2007; Sherpa et al., 2008). Nevertheless, because the Müller glia are the main source of regenerated retinal neurons, complete ocular excision prevents regeneration (Mensinger and Powers, 2007).

In adult teleosts, although there is robust regeneration after extensive retinal injury, the time required for regeneration and functional recovery depends on the extent of injury. For example, the differences in regeneration and rewiring were examined after surgical extirpation of ∼25, 50, and 75% of the adult retina. This work found that after surgical extirpation of 25% of the retina, 17 weeks (120 days) are required for regeneration and reestablishment of lamination and 25 weeks (180 days) for functional recovery. Additional extirpation lengthened the time and extent of recovery for both lamination and functional recovery (Mensinger and Powers, 2007). Similar results were observed in adults after pharmacological damage and death of all retinal neurons (induced by high doses of intraocular ouabain). In this study functional recovery started 5 weeks after the injury with further improvement by weeks 7 – 10, albeit with decreased sensitivity (Mensinger and Powers, 1999; Lindsey and Powers, 2007).

In adult zebrafish, the extent and time course of retinal regeneration and rewiring is also dependent on the injury. Extensive pharmacological damage of all retinal layers with ouabain leads to regeneration, and the newly formed cells are capable to reorganize into the three distinct nuclear layers by week 3 after injury. After 14 weeks, the regenerated retinas are well laminated (clear nuclear and plexiform layers), the optic nerve has regrown, and there is functional recovery (Sherpa et al., 2008). Interestingly, with pharmacological damage (using lower doses of ouabain) that spares photoreceptors and Müller glia but still induces a loss of cells in the INL and GCL, regeneration is faster, with significant recovery of function after only 8 weeks (Sherpa et al., 2014; McGinn et al., 2018). In this lesion paradigm, rewiring of a specific (but heterogeneous) subset of regenerated BCs was closely examined. The regenerated BCs had largely normal morphology, and, as a population, were able to reproduce the diversity of connectivity patterns observed in the surviving photoreceptors, with only a few errors in lamination or abnormal dendritic or axonal arborization, again emphasizing the robust regeneration of zebrafish (McGinn et al., 2018). Nevertheless, in the context of extensive damage, regeneration in adult teleosts is far from perfect. Several structural defects are common including areas with defects in the formation or absence of plexiform layers, disorganization of nuclear layers, presence of cells in the incorrect layer (e.g., RGCs in INL), failure to reestablish the photoreceptor mosaic, formation of photoreceptor rosettes, overproduction of neurons, and the generation of cell types that were not initially damaged (Raymond et al., 1988; Hitchcock et al., 1992; Cameron, 2000; Vihtelic Thomas and Hyde David, 2000; Stenkamp et al., 2001; Stenkamp and Cameron, 2002; Sherpa et al., 2008; Powell et al., 2016).

More recently, the genetic tractability of zebrafish has enabled researchers to damage specific retinal cell types and study their rewiring after regeneration. This work was accomplished by using the recently developed nitroreductase–metronidazole (NTR–MTZ) system. For this method, transgenic zebrafish are created expressing the NTR gene (*nfsb*) under the control of a cell-specific promoter. When these transgenic zebrafish are treated with the compound MTZ, the NTR converts MTZ into a cytotoxic compound. Because this compound does not diffuse to neighboring cells, the resulting ablation is restricted to the NTR-expressing cells. Importantly, this process is reversible, and removal of MTZ solution makes it possible to examine regeneration and rewiring. To date, the NTR-MTZ system has been used to selectively ablate rods, and specific subtypes of cones, bipolar cells and glial cells (Zhao et al., 2009; Ariga et al., 2010; Montgomery et al., 2010; Fraser et al., 2013; D’Orazi et al., 2016; Hagerman et al., 2016; White et al., 2017). Importantly, several of these studies demonstrated that other cells in the retina that did not express NTR were not ablated after MTZ application. In addition, these treatments did not affect the surrounding retinal architecture (Zhao et al., 2009). This highlights the specificity and power of the NTR-MTZ system.

After genetic ablation and removal of MTZ, in each instance the targeted cells regenerated after several days, although the exact time-course varied depending on the ablated cell type and the age of zebrafish treated. For example, after using the NTR–MTZ to completely ablate rods in adult zebrafish, newly generated rods were identified within a week after removal of MTZ, and repopulation of rods attained pre-injury levels within 4 weeks (Montgomery et al., 2010) – a very similar time course required for the regeneration of cones in adults (Raymond et al., 2006; Bernardos et al., 2007). In larvae, the regeneration of cells occurs at a faster timescale. For example, after ablation of rods using the NTR-MTZ system in 5 dpf larvae, newly formed rods attained control levels in just 6 days (White et al., 2017). Similarly, cones ablated between 4 and 6 dpf regenerate in 7 – 10 days (Fraser et al., 2013; Yoshimatsu et al., 2016) and BCs ablated at 7 dpf regenerate in 13 days (D’Orazi et al., 2016).

In the majority of these studies, regeneration was confirmed morphologically. In a subset of studies, after regeneration, the analysis was extended to include behavior or rewiring. For example, in one study, either the UV- or S-cones were ablated (in 7 dpf larvae) and the optomotor response (OMR) (see below) was assayed after ablation and following UV- or S-cone regeneration respectively (Hagerman et al., 2016). The OMR was reduced immediately after ablation of either UV- or S-cones. Surprisingly, while the OMR recovery took 4 days for the UV-cone ablation, the OMR recovered in just 1 day following S-cone ablation, before new S-cones were produced. These differences in behavioral recovery suggest that there may be a capacity for plasticity amongst the remaining cells, used to compensate for the ablated cells during the recovery phase. It is possible that this short-term plasticity relies on activity from other cone subtypes and/or on synaptic remodeling. Evidence for such synaptic remodeling has been reported in a parallel study (Yoshimatsu et al., 2016). In this study, a subtype of horizontal cell (H3) that normally connects preferentially to UV- and S-cones, was able to reconnect to UV-cones after UV-cone specific ablation and regeneration. Nevertheless, if UV-cone regeneration was delayed, the H3 made additional contacts with S-cones and even M- and L-cones, suggesting functional compensation at the level of rewiring. Another recent study examined rewiring after selective loss of a subpopulation of BC using the NTR-MTZ system in 5 dpf larvae (D’Orazi et al., 2016). Thirteen days after the ablation of these BC, the majority of regenerated BC were morphologically normal but the rewiring did not fully recapitulate development, with a relative loss of selectivity for specific cone subtypes. Additionally, BC axons contained significantly more synapses.

As a whole, work in this field proves that retinal regeneration in zebrafish is a robust process, but also suggests that some of the developmental cues required to refine synapse number or proportion of photoreceptor subtypes innervated may not be present during regeneration. In the future, it will be important to further understand what cues are present during development that enable photoreceptors to wire into different retinal circuits. It will be especially important to understand how specific photoreceptor subtypes recognize the different bipolar- and horizontal-cell subtypes, and the factors required for the formation of these synapses. It will also be important to recognize and examine the developmental and environmental differences between larval and adult zebrafish retinas. This knowledge will provide a comprehensive understanding of the differences between development and regeneration, between wiring, rewiring and remodeling, and likely uncover manipulations that could be used to modify or refine rewiring in the context of treatments for RD.

## The Zebrafish Toolkit for the Study of Retinal Development and Regeneration

There are numerous factors that have established zebrafish as a valuable model organism for the study of human disease including rapid development, large clutch sizes, ease of maintenance, genetic conservation, accessibility to genetic manipulations, and optical transparency of embryos. In addition to these advantages, we have described several examples of how zebrafish has been a useful model to investigate retinal development and regeneration. To aid in these studies, multiple tools have been developed that have direct application for the study of retina in zebrafish. We have summarized *some* of these tools here as a convenient reference.

### Imaging Retinal Cells and Their Connectivity

**FIGURE 3:**
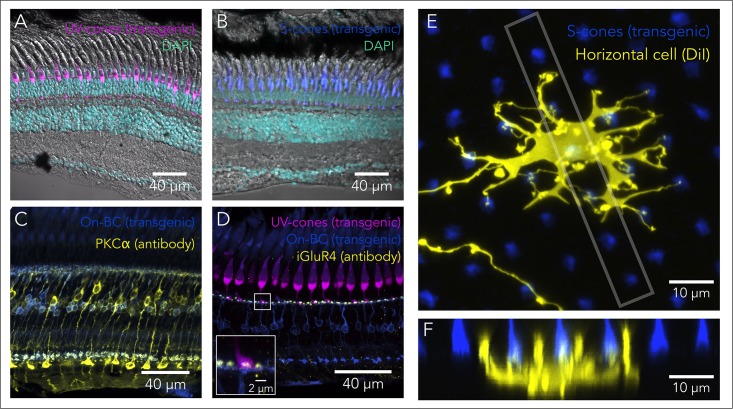
Tools to study retinal circuits. **(A)** Confocal image of the UV-cone reporter line *Tg(sws1:GFP)^kj9^* retina (magenta), overlaid with DAPI nuclear staining (cyan) and a transmitted DIC image. **(B)** Confocal image of the S-cone reporter line *Tg(-3.5opn1sw2:EGFP)^k11^* retina (blue), overlaid with DAPI nuclear staining (cyan) and a transmitted DIC image. **(C)** Bipolar cell labeling using PKCα immunolabeling (yellow) and the On-bipolar reporter line *Tg(grm6b:EGFP)^zh1^* (blue). Rod-contacting bipolar cells are brightly labeled by the PKCα antibody, while another subset of bipolars is more dimly labeled. Some of these are doubly labeled with the transgenic line. **(D)** Immunolabeling of off-bipolar synapses with photoreceptors using an antibody against the inotropic glutamate receptor type 4 (*gria4*), in the background of a double transgenic reporter line *Tg(sws1:GFP)^kj9^* (magenta) and *Tg(grm6b:EGFP)^zh1^* (blue). Inset shows that the punctate labeling in the IPL overlaps with the synaptic terminals of cones and the bipolar cell dendrites. **(E)** Sparse labeling of horizontal cells using DiI (yellow) in the background of the S-cone reporter line *Tg(-3.5opn1sw2:EGFP)^k11^*. Image corresponds to a maximal intensity projection of a confocal stack, where synaptic contacts between the horizontal cell and S-cones are apparent. **(F)** Maximal intensity projection of an orthogonal view restricted to the box gray in **(E)**, highlighting the invaginations of horizontal processes into the cone synaptic terminals. This particular projection is oriented through a row of UV- and S-cones and the bigger horizontal processes most likely correspond to invaginations into UV-cone terminals.

Currently there are established transgenic lines that make it straightforward to visualize each cell subtype within the retina, as well as the lamination and precise wiring of these cells (**Table 1**). For example, taking advantage of the specificity of opsin expression in the different photoreceptor subtypes, promoters from each opsin have been utilized to create transgenic lines that label rods (rods: *rho*) (Fadool, 2003) and each of the four cone subtypes (UV-cones: *opn1sw1*; S-Cones: *opn1sw2*; M-Cones: *opn1mw2*; L-Cones: *opn1lw1)* (Takechi et al., 2003, 2008; Tsujimura et al., 2007, 2010) (**Figures 3A,B**). Additionally, multiple lines exist to label horizontal and BC. Within the retina, horizontal cells can be specifically labeled exploiting the promoter for *connexin 55.5* (Weber et al., 2014; Klaassen et al., 2016), or using an enhancer-trap line that labels a combination of horizontal and amacrine cells (Torvund et al., 2017). BC represent a more diverse cell class. On-BC can be labeled using the promoter for *grm6b*, a metabotropic glutamate receptor that is key for the detection of glutamate release by photoreceptors (Glasauer et al., 2016) (**Figure 3C**), and different subtypes of BC have been labeled with enhancer-trap lines (D’Orazi et al., 2016), or using promoters for transcription factors (Vitorino et al., 2009) or for other bipolar-specific proteins (Schroeter et al., 2006). The promoter for *gfap* (glial fibrillar acidic protein) can be used to label Müller glia (Raymond et al., 2006; Bernardos et al., 2007). Additionally, lines that use the promoter for *mpeg1* label all macrophages (Ellett et al., 2011), allow visualization of the retinal microglia and macrophages in both the normal and regenerating retina (Mitchell et al., 2018).

**Table 1:** Toolkit for the study of retinal development and regeneration: transgenic lines.

Target cell types	Genotype	Description	ZFIN ID	Reference
**Reporter lines**				
**Photoreceptors**				
Rods	*Tg(XlRho:EGFP)^fl1^*	Promoter of rhodopsin from *Xenopus* drives the expression of GFP in rods	ZDB-ALT-080517-1	Fadool, 2003
Rods	*Tg(rho:EGFP)^kj2^*	Promoter of rhodopsin drives expression of GFP in rods	ZDB-ALT-060830-4	Hamaoka et al. (2002)
Cones (all subtypes)	*Tg(-3.2gnat2:EGFP)^ucd1^*	Promoter of cone transducin alpha subunit drives the expression of GFP in all cone subtypes	ZDB-ALT-070829-1	Smyth et al., 2008
UV-cones	*Tg(sws1:GFP)^kj9^*	Promoter of UV-opsin drives the expression of GFP in UV-cones	ZDB-ALT-080227-1	Takechi et al., 2003
S-cones	*Tg(-3.5opn1sw2:EGFP)^k11^*	Promoter of S-opsin region drives the expression of GFP in S-cones	ZDB-FISH-150901-14019	Takechi et al., 2008
S-cones	*tg(sws2:mCherry)^ua3011^*	Promoter of S-opsin drives expression of mCherry in S-cones	ZDB-ALT-130819-1	Duval et al., 2013
S-cones	*Tg(opn1sw2:mCherry)^mi2007^*	Promoter of S-opsin drives expression of mCherry in S-cones	ZDB-ALT-120921-1	Salbreux et al., 2012
M-cones	*Tg(opn1mw2:EGFP)^kj4^*	Promoter of M-opsin drives the expression of GFP in M-cones	ZDB-ALT-071206-2	Tsujimura et al., 2007
L-cones	*Tg(-0.6opn1lw1-cxxc1:GFP)^kj19d^*	Promoter of L-opsin genes drives the expression of GFP	ZDB-ALT-110519-13	Tsujimura et al., 2010
L-cones	*Tg(thrb:Tomato)^q22^*	Promoter of thyroid hormone receptor β drives the expression of tdTomato in L-Cones	ZDB-FISH-150901-15085	Suzuki et al., 2013
**Horizontal cells**				
All horizontal cells	*Tg(lhx1a:EGFP)^pt303^*	Promoter of LIM Homeobox 1 drives expression of GFP in horizontal cells	ZDB-ALT-100323-3	Rao et al., 2017
All horizontal cells	*Tg(cx55.5:MA-GFP)^zf524^*	Promoter of connexin 55.5 drives the expression of GFP in all horizontal cells	ZDB-ALT-150319-1	Weber et al., 2014
All horizontal cells	*Tg(cx55.5:EGFP)^nhi1^*	Promoter of connexin 55.5 drives the expression of GFP in all horizontal cells	ZDB-ALT-180119-1	Klaassen et al., 2016
Horizontal cell subtype (H4)	*Tg(cx52.7:EGFP)^nhi2^*	Promoter of connexin 52.7 drives the expression of GFP in H4 horizontal cells	ZDB-ALT-180119-2	Klaassen et al., 2016
Horizontal cell subtype (H1)	*Tg(cx52.9:EGFP)^nhi3^*	Promoter of connexin 52.9 drives the expression of GFP in H1 horizontal cells	ZDB-ALT-180119-3	Klaassen et al., 2016
Horizontal cell subtypes (H1,H4)	*Tg(cx52.6:EGFP)^nhi4^*	Promoter of connexin 52.6 drives the expression of GFP in H1 and H4 horizontal cells	ZDB-ALT-180119-2	Klaassen et al., 2016
Horizontal cell subtype	*Tg(hsp70l:EGFP)^nds1^*	Enhancer trap drives the expression of GFP in horizontal and several subtypes of amacrine cells	ZDB-ALT-170512-1	Torvund et al., 2017
**Bipolar cells**				
On Bipolar Cells	*Tg(grm6b:EGFP)^zh1Tg^*	Promoter of metabotropic glutamate receptor 6b drives the expression of GFP in bipolar cells, and subtypes of amacrine and ganglion cells	ZDB-ALT-160819-2	Glasauer et al., 2016
Bipolar cells (subtypes)	*Tg(GAL4-VP16,UAS:EGFP)^ub43^*	Enhancer trap drives the expression of GFP in subtypes of On- and Off-bipolar cells	ZDB-ALT-100201-1	D’Orazi et al., 2016
Bipolar cells (subtypes)	*Tg(vsx1:GFP)^nns5^*	Promoter of visual system homeobox 1 drives the expression of GFP in subtypes of bipolar cells	ZDB-ALT-061204-4	Vitorino et al., 2009
Bipolar cells (subtypes)	*Tg(chx10:loxP-dsRed-loxP-GFP)^nns3^*	Promoter ofvisual system homeobox 2 drives the expression of GFP in subtypes of bipolar cells, and Müller glia	ZDB-ALT-090116-1	Vitorino et al., 2009
On-bipolar cells (subtypes)	*Tg(nyx:Gal4-VP16)^q16a^*	Promoter of nyctalopin drives the expression of Gal4 on a subset of On-bipolar cells	ZDB-ALT-071129-2	Schroeter et al., 2006; McGinn et al., 2018
**Müller glia**				
Müller glia	*Tg(gfap:GFP)^mi2001^*	Promoter of glial fibrillary acidic protein drives the expression of GFP	ZFIN ID: ZDB-ALT-060623-4	Raymond et al., 2006
Müller glia	*Tg(gfap:nGFP)^mi2004^*	Promoter of glial fibrillary acidic protein drives the expression of nucleus-localized GFP	ZDB-ALT-070830-1	Bernardos et al., 2007
**Immune cells**				
Microglia and macrophages	*Tg(mpeg1:EGFP)^gl22^*	Promoter of macrophage-expressed gene 1 drives the expression of GFP	ZDB-ALT-120117-1	Ellett et al., 2011; Mitchell et al., 2018
Microglia and macrophages	*Tg(mpeg1:mCherry)^gl23^*	Promoter of macrophage-expressed gene 1 drives the expression of mCherry	ZDB-ALT-120117-2	Ellett et al., 2011; Mitchell et al., 2018
**Lines for selective ablation (NTR-MTZ)**				
Rods	*Tg(zop:nfsb-EGFP)^nt19^*	Promoter of rhodopsin drives the expression of nitroreductase-GFP fusion in rods	ZDB-ALT-100323-4	Montgomery et al., 2010
UV-cones	*Tg(opn1sw1:KALTA4)^ua3139^*	Promoter of UV-opsin drives the expression of KalTA4, which binds to a separate transgene and drives expression of a nitroreductase and mCherry fusion protein in UV-cones	ZDB-ALT-160901-14	Hagerman et al., 2016
UV-cones	*Tg(opn1sw1:NTR-mCherry)^q28^*	Promoter of UV-opsin drives the expression of a nitroreductase and mCherry fusion protein in UV-cones	ZDB-ALT-160425-1	Yoshimatsu et al., 2016
S-cones	*Tg(opn1sw2:NTR-mCherry)^q30^*	Promoter of S-opsin drives expression of nitroreduactase-mCherry fusion in S-cones	ZDB-ALT-160425-3	D’Orazi et al., 2016; Yoshimatsu et al., 2016
Bipolar cells	*Tg(UAS-E1b:NfsB-mCherry)^c264^*	Enhancer trap drives the expression of a nitroreductase and mCherry fusion protein in a subset of bipolar cells	ZDB-ALT-070316-1	Zhao et al., 2009; D’Orazi et al., 2016
**Lines for functional imaging**				
Photoreceptors and Bipolar Cells	*Tg(-1.8ctbp2:Rno.Syp-GCAMP)^lmb3^*	Promoter of ribeye drives synaptically localized GCaMP2 in photoreceptors and bipolar cells	ZDB-ALT-120320-5	Dreosti et al., 2009
Photoreceptors and Bipolar Cells	*Tg(-1.8ctbp2a:Rno.Syp-GCaMP6)^uss1^*	Promoter of ribeye drives synaptically localized GCaMP6 in photoreceptors and bipolar cells	ZDB-ALT-161010-18	Johnston et al., 2014
Photoreceptors and Bipolar Cells	*Tg(-1.8ctbp2:SYPHY)^lmb2^*	Promoter of ribeye drives synaptic phluorin in photoreceptors and bipolar cells	ZDB-ALT-120320-4	Odermatt et al., 2012
Müller glia	*Tg(gfap:Eco.GltL-cpEGFP)^cu3313^*	Promoter of glial fibrillary acidic protein drives the expression of iGluSnfR	ZDB-ALT-170404-12	MacDonald et al., 2017


Most of these lines allow high-resolution imaging of not only the lamination but also the synaptic terminals of these distinct cells types (Noel and Allison, 2018) (**Figure 3D**). For example, in some of these transgenic lines, the connections of photoreceptors have been used to accurately track wiring during development (Yoshimatsu et al., 2016) or rewiring after regeneration (D’Orazi et al., 2016). They can also be adapted for live imaging, allowing connections to be dynamically tracked during circuit formation or during the integration of regenerated neurons into functional retinal circuits (Ariga et al., 2010; Duval et al., 2013). Using the same promoters, several transgenic lines have been developed to express the NTR gene (*nfsb)* and ablate specific subsets of retinal cells (see above, **Table 1**). This approach has enabled the study of regeneration and rewiring after selective ablation (Zhao et al., 2009; D’Orazi et al., 2016; Yoshimatsu et al., 2016; McGinn et al., 2018; Noel and Allison, 2018). Lastly, it is straightforward to perform sparse labeling to capture the fine detail of individual retinal neurons and their contacts during development or regeneration, by using transient expression in larvae (with the same promoters leveraged for transgenic lines) (Klaassen et al., 2016; Yoshimatsu et al., 2016). Alternatively, inorganic fluorescent dyes like DiI can be used in larvae or in adults (**Figures 3E,F**) (Connaughton et al., 2004; Li et al., 2009; Li et al., 2012).

While transgenic lines represent powerful tools to visualize cells and processes within the retina *in situ*, antibodies against specific markers have been identified that can also be exploited to label subsets of retinal cells (**Table 2**). In addition to antibodies, cones can also easily be labeled using fluorescently-tagged *peanut agglutinin* (or PNA), a lectin protein that binds to the cone sheath (Hageman and Johnson, 1986; Shi et al., 2017). In combination with the transgenic lines outlined above, these labels can be used to visualize multiple types of cells simultaneously within the retina (**Figure 3C**). In addition to simply marking specific retinal cell types, antibodies against synaptic markers are of particular usefulness to characterize retinal wiring. In photoreceptors most presynaptic markers label components of the photoreceptor ribbon synapse, like Ribeye or Syntaxin, or adjoining structures like the voltage-gated calcium channels or the synaptic vesicles (Huang et al., 2012; Lv et al., 2012; Daniele et al., 2016). Postsynaptically, photoreceptor synapse markers include: components of the postsynaptic density itself (e.g., MAGUK) and glutamate receptors (*grm6* for ON-bipolars, *gria4* for OFF-bipolars, *gria2* for horizontal cells) (Yazulla and Studholme, 2001) (**Figure 3D**). These synaptic markers are extremely important for understanding the correct development of synapses or the correct rewiring of photoreceptors after regeneration. For example, alterations in the synapses between cones and Off-BC caused by mutations in *pappaa*, a protein recently identified in a behavioral screen, were identified by labeling the photoreceptor synaptic vesicles, but could not be seen by labeling specific cell-types, as retinal lamination was not altered (Miller et al., 2018).

**Table 2:** Toolkit for the study of retinal development and regeneration: antibodies and fluorescent labeling.

Labeled cell types or structures	Antibody	Antigen	Manufacturer (Catalog number) Species	ZFIN ID	Reference
Photoreceptors					
Rods	1D4	C-terminus of bovine rhodopsin	Sigma-Aldrich (MAB5356) Abcam (ab5417) Mouse monoclonal	ZDB-ATB-110114-2	Linder et al., 2011
Rods	*gnat1*	Human transducin alpha subunit	ProteinTech (55167-1-AP) Rabbit polyclonal	ZDB-ATB-151005-5	Liu et al., 2015
Cones	Recoverin	Human recoverin	Millipore (AB5585) Rabbit polyclonal	ZDB-ATB-151016-2	Solin et al., 2014
	Peanut agglutinin	Cone extracellular matrix	Molecular Probes (L21409)		Hageman and Johnson, 1986
M- and L-cones	*zpr1*	Arrestin3a	ZIRC (AB_10013803) Mouse Monoclonal	ZDB-ATB-081002-43	Ile et al., 2010
	1D4	C-terminus of bovine rhodopsin	Abcam (ab5417) Mouse monoclonal	ZDB-ATB-121128-10	Yin et al., 2012
Photoreceptor synapses	*ribeyeA*	Ribeye A	Teresa Nicolson Laboratory OHSU Rabbit Polyclonal	ZDB-ATB-120504-2	Randlett et al., 2013
	*SV2*	Synaptic Vesicle glycoprotein 2	DSHB, Iowa, United States Mouse monoclonal	ZDB-ATB-081201-1	Huang et al., 2012
	syntaxin3	Syntaxin 3	Synaptic Systems (110033) Rabbit polyclonal	ZDB-ATB-160428-2	Lin et al., 2016
Horizontal cells					
	GluR2	Glutamate receptor type 2	Millipore (MAB397) Mouse monoclonal	ZDB-ATB-151118-1	Yazulla and Studholme, 2001
	GAD67	Glutamate decarboxylase 67 kDa isoform	Chemicon International Inc. (AB108) Rabbit Polyclonal	ZDB-ATB-100903-8	Yazulla and Studholme, 2001
Bipolar cells					
Bipolar cells (subset)	PKCα	Protein kinase C alpha subunit	Sigma (P4334) Rabbit polyclonal	ZDB-ATB-090223-3	Yazulla and Studholme, 2001; Moshiri et al., 2008
On-bipolar cell subtype	mGluR1	metabotropic glutamate receptor 1 alpha subunit	Millipore (AB1551) Rabbit polyclonal	ZDB-ATB-100810-1	Yazulla and Studholme, 2001
On- and Off-bipolar cells	anti-GABAa α3	GABA receptor alpha subunit 3	Alomone Labs (#AGA-003) Rabbit polyclonal	ZDB-ATB-100903-6	Yazulla and Studholme, 2001
On Bipolar Cells	PKCβ1 (C-16)	Protein kinase C beta subunit 1	Santa Cruz Biotechnology, sc-209 Rabbit polyclonal	ZDB-ATB-120614-1	Glasauer et al., 2016
On-bipolar cell dendrites	Pan-Maguk	Membrane-associated guanylate kinases	Neuromab (75-029, clone K28/86) Mouse monoclonal	ZDB-ATB-120504-1	Randlett et al., 2013
Off-bipolar cell dendrites	gria4	Ionotropic glutamate receptor type 4	Millipore (AB1508) Rabbit polyclonal	ZDB-ATB-100810-2	Yazulla and Studholme, 2001
Amacrine cells					
Starburst	ChAT	Human placental choline acetyltransferase	Millipore, AB144P, Goat polyclonal	ZDB-ATB-081017-3	Glasauer et al., 2016
GABAergic	GAD65/67	Glutamic acid decarboxylase 65 kDa/67 kDa	Abcam (Ab11070) Rabbit polyclonal	ZDB-ATB-090617-2	Glasauer et al., 2016
Dopaminergic	TH	Tyrosine hydroxylase	Immunostar, Inc. (22941) Mouse monoclonal	ZDB-ATB-081017-8	Glasauer et al., 2016
Müller glia					
	GS	Glutamine Synthase	Millipore (mab302) Mouse monoclonal	ZDB-ATB-081009-5	Randlett et al., 2013
	*gfap*	Glial Fibrillary Acidic Protein	ZIRC (zrf-1) Mouse monoclonal	ZDB-ATB-081002-46	Solin et al., 2014
	*glt1*	Glutamate transporter 1 (glial)	Millipore (AB1783) Guinea pig polyclonal	ZDB-ATB-100916-6	Yazulla and Studholme, 2001
Ciliary Marginal Zone	PCNA	Anti-Proliferating Cell Nuclear Antigen	Santa Cruz Biotechnology, sc-56 Mouse monoclonal	ZDB-ATB-081121-4	Inoue and Wittbrodt, 2011
Bipolar, horizontal and amacrine cells	isl1	Islet 1	DSHB, Iowa, United States (40.3A4) Mouse monoclonal	ZDB-ATB-081124-3	Zhang et al., 2012
Bipolar, horizontal, amacrine and ganglion cells	HuC/HuD	ELAV like neuron-specific RNA binding protein 3 and 4	Invitrogen (A-21271) Mouse monoclonal	ZDB-ATB-081003-2	Randlett et al., 2013


### Functional and Behavioral Methods to Assess the Zebrafish Retina

One of the most exploited assays for visual function in humans and many animal models are electroretinograms (ERG). ERG measure bulk electrical signals produced by the whole retina in response to light stimulation and has been adapted to both zebrafish larvae (Nelson and Singla, 2009; Chrispell et al., 2015) and adults (Hughes et al., 1998). Through analysis of the different ERG waves the overall activity of photoreceptors and BC can be evaluated. Using well-designed stimuli or pharmacological agents, other properties like the kinetics of photoreceptor adaptation can also be measured (Korenbrot et al., 2013). Additionally, ERG signals have a spectral signature based on the signals generated by specific subsets of photoreceptor and their downstream partners. These signatures can be utilized to isolate the contributions of each element in different conditions (Nelson and Singla, 2009). ERG measurements have been used in the adult to demonstrate that the retina can recover function after damage and subsequent regeneration (McGinn et al., 2018). In the future ERG could be used both in larvae and adults after NTR-MTZ ablation of specific cells, to assess the functional recovery of retinal processing during regeneration.

While the ERG can provide a powerful readout of retinal activity, functional imaging using genetically encoded calcium sensors has also been developed to measure response in visual centers, especially in the tectum (Förster et al., 2017). Currently these approaches remain challenging in the retina due to the RPE which creates an optical barrier, making it difficult to image directly through the eye. It is possible to use these indicators to image through the lens in adults (Duval et al., 2013). Also, in larvae some transparency can be achieved using PTU to inhibit melanophore production, but this treatment may alter visual function (Antinucci and Hindges, 2016). Better imaging has been achieved with mutant lines that genetically remove the different classes of pigmented cells (White et al., 2008; Antinucci and Hindges, 2016). In the future, additional functional imaging using calcium sensory in photoreceptors along with newly developed neurotransmitter sensors, like iGluSnfR (Marvin et al., 2013), will be an important *in vivo* approach to assess pre- and post-synaptic function with in developing and regenerating retinal circuits. (Zhang et al., 2016; MacDonald et al., 2017).

In addition to electrically or optically recording the activity of cells within the retina, there are many well characterized visual behaviors that can be used to evaluate retinal function. The *optokinetic response* is an extremely robust behavioral assay, where a visual stimulus of moving stripes is tracked by eye movements. This behavior is already present and reliable by 5 dpf and requires minimal equipment to setup (Brockerhoff et al., 1995; Neuhauss et al., 1999; Neuhauss, 2003). In the related *optomotor response*, tracking of moving stripes is followed by swimming in the same direction as the stimulus (Neuhauss et al., 1999; Neuhauss, 2003). This assay has been used to characterize the overall recovery of vision after photoreceptor ablation (Hagerman et al., 2016). At around 5 dpf, larvae also start hunting for small prey using visual cues, another visual behavior that can be quantified (Borla et al., 2002; Gahtan et al., 2005; McElligott and O’Malley, 2005). Larvae also innately exhibit phototaxis and photoavoidance (Brockerhoff et al., 1995; Orger and Baier, 2005; Burgess et al., 2010), and an escape response in response to sudden decreases in illumination (Burgess and Granato, 2007).

Adult zebrafish also exhibit an *escape response* to threatening objects, characterized by rapid turning and swimming away from the threat. The escape response can be elicited by placing fish in a clear tank with a central pole that serves for hiding, and an external rotating drum with a single black stripe to act as a threatening stimulus. Use of this assay allowed to measure behavioral rod and cone thresholds and the time course of photoreceptor adaptation and as part of the screening in a forward-genetic screen for visual mutations (Li and Dowling, 1997; Li and Dowling, 2000). Zebrafish and other teleosts determine their body position using a combination of their sense of balance and the source of illumination, which in their natural environment tends to come from above. Thus, they tend to tilt their bodies such that their backs are turned against the source of illumination (*dorsal light response* or DLR). Tilt can be induced by uneven-illumination between the two eyes (e.g., side illumination) (Silver, 1974; Neuhauss, 2003) or by unilateral ocular damage, where recovery of a normal tilt is a sign of functional recovery (Mensinger and Powers, 1999; Lindsey and Powers, 2007; Mensinger and Powers, 2007; Sherpa et al., 2014). In zebrafish, information from the vestibular system is capable of overriding visual input, so that the DLR is only apparent when the vestibular system has been damaged (Nicolson et al., 1998) or when fish are placed head down in a tightly fitting tube (Neuhauss, 2003).

In captivity, zebrafish are conditioned to move toward the front of the tank and wait for food whenever a person approaches. This conditioned learning can be exploited to test for visual function (*place preference test*) (Sherpa et al., 2014). A very similar test has been recently used in cichlids to demonstrate their ability to truly discriminate colors (Escobar-Camacho et al., 2017) (**Table 3**).

**Table 3:** Toolkit for the study of retinal development and regeneration: electroretinograms and visually guided behaviors.

Visual assay	Description	References
Electroretinogram (ERG)	Measurement of the changes in the bulk electrical activity produced by the retina when stimulated with light. Can be measured in both larvae and adults.	Hughes et al., 1998; Chrispell et al., 2015
Optokinetic reflex (OKR)	Rotational eye movements track moving targets, usually white and black stripes in a rotating drum, are followed by saccades that reset the eye position.	Clark, 1981; Neuhauss et al., 1999; Neuhauss, 2003
Optomotor response (OMR)	Swimming in the direction of a moving stimuli.	Clark, 1981; Neuhauss, 2003
Prey/Small object tracking	Larvae hunt for paramecia by making stereotyped swimming movements to orient themselves before swimming forward to capture their prey	Borla et al., 2002; Gahtan et al., 2005; McElligott and O’Malley, 2005
Phototaxis and Photoavoidance	Swim movements towards or away from light sources	Brockerhoff et al., 1995 Orger and Baier, 2005; Burgess et al., 2010
Escape response to sudden decreases in illumination	Larval zebrafish exhibit escape responses (“O-bends”) to sudden decreases in illumination	Miller et al., 2018
Escape response to dark objects	Adult zebrafish avoid moving dark objects by abruptly changing the direction of swimming	Li and Dowling, 1997
Dorsal light reflex (DLR)	Zebrafish tilt their bodies to turn their backs towards the source of illumination by keeping equal input in both eyes, but vestibular information can override this reflex. Reflex can be made apparent by placing fish head down in a tightly fitting tube.	Nicolson et al., 1998; Neuhauss, 2003
Place preference	Captive zebrafish are conditioned by feeding routines to move toward front of the tank when they visually detect a person approaching.	Sherpa et al., 2014


Interestingly, some of these behaviors seem to rely on only into small subsets of retinal circuits. For example, the OKR seems to be mainly driven by M- and L-cone signals (Orger and Baier, 2005), while the tracking of small dots, at least in goldfish, depends mainly on M-cone signals (Gehres and Neumeyer, 2007). Similarly, photoavoidance is robustly driven by UV-light, and presumably UV-cone signals (Guggiana-Nilo and Engert, 2016). From previous work that has shown fast functional OKR recovery after S-cone ablation even before regeneration and rewiring occur (Hagerman et al., 2016), it is clear that not enough is known about the function, recovery and plasticity of circuits in the retina. Together, behavioral assays along with NTR-mediated ablation of a given cone subtype, could be used to further expand our understanding of these circuits, and the functional consequences of regeneration and rewiring of the different retinal circuits.

### Forward and Reverse Genetic Approaches

Zebrafish forward genetic screens are extremely powerful, and have been successfully used to uncover novel genes that are involved in photoreceptor function and RD (Brockerhoff and Fadool, 2011). It is likely that any additional screening that relies on alterations in visual behaviors will continue to uncover new genes that affect photoreceptor function or cause RD. As more causes of RD continue to be unveiled, we now understand that there are limited number of converging pathways that eventually lead to photoreceptor degeneration, including: classic apoptosis, oxidation, activation of proteolytic pathways, misbalance in intracellular levels of cGMP and calcium, and epigenetic regulation (Trifunovic et al., 2012). With this knowledge, reverse-genetic approaches using CRISPR-mediated gene editing to target previously undescribed components within these pathways are likely to be extremely useful in the future. Recent studies in zebrafish have already demonstrated that zebrafish can be used as a platform to rapidly perform genetic screens using CRISPR (Varshney et al., 2015, 2016; Shah et al., 2016). In addition to genetic screens, similar to what has been done in fin and hair-cell regeneration studies (Mathew et al., 2007; Namdaran et al., 2012), pharmacology-based screening could be used to isolate novel compounds with the ability to promote or prevent photoreceptor regeneration. Similar screens could also be accomplished using behavioral assays, evaluating the recovery of visual function after regeneration. Some of the success of such screens in hair cells of the lateral line stems from the fast regeneration times (∼2 days) and the small number of cell types that have to be regenerated. In paradigms of regeneration after extensive damage (surgical of pharmacological) in adults, the long regeneration times (8–14 weeks) and the diversity of cell types that need to be regenerated might present insurmountable hurdles for screens. Nevertheless, photoreceptor regeneration occurs within 4 weeks in adults and in ∼10 days in larvae after cell-specific ablations, opening up the possibility to carry out such screens.

While the majority of regeneration studies in zebrafish have focused on regenerating damaged cells, for functional recovery after regeneration, it is imperative that new cells integrate appropriately into their specific retinal circuits. Our current knowledge on how retinal circuits in the outer retina form during development and after regenerations is limited. Studies into these processes suggest that rewiring after regeneration is not a complete recapitulation of development. First of all, during development retinal cells are derived from retinal progenitors and retinal circuits assemble properly even in the absence of Müller glia (Williams et al., 2010). In contrast, during regeneration, Müller glia are the principal source of new retinal cells. Second, not all the transcription pathways that are active during development are reactivated during regeneration (Veldman et al., 2007; Sherpa et al., 2014). Third, there seems to be more plasticity and a greater capacity for compensation. It appears that during rewiring, at least in larvae, maintaining inputs and outputs is more important than the absolute selectivity of connections (D’Orazi et al., 2016; Yoshimatsu et al., 2016). In adult zebrafish, after pharmacological ablation of bipolar, amacrine and ganglion cells (but survival of photoreceptors) and their subsequent regeneration, various subtypes of BCs seem to be able to recapitulate the diversity of connectivity that is found in uninjured eyes, but as a population, selectivity for photoreceptors seems to be restored (McGinn et al., 2018). Further investigation is required to explain the disagreement between these studies since there are many differences including ablation technique (NTR-MTZ vs. ouabain), age of ablation (larvae vs. adults), time between ablation and assessment of connectivity (1–2 weeks vs. 8 weeks) and subtypes of cells studied. Yet, this raises interesting questions: is the capacity for compensation only present in larvae and lost in the adult? or, is compensation only present in the initial phase after regeneration and normal selectivity of connections reestablished over time?

It is interesting to note that selectivity is not lost during rewiring, it is just more permissive, and it is very likely that the same molecules that allow recognition between retinal cell types are used in both development and regeneration. Only a handful of cell-adhesion and synaptic molecules are known to be necessary for the formation of synaptic contacts between photoreceptors and downstream retinal cells (Zhang et al., 2017; Miller et al., 2018). Some of these molecules are key across all photoreceptors, while others are specific to rods (Cao et al., 2015; Wang et al., 2017) or to cones (Sarria et al., 2018; Ueno et al., 2018). To date, the molecular mechanisms involved in the recognition between specific photoreceptor subtypes and their synaptic partners (horizontal and bipolar cells) are not known. Any of the genetic screening tools mentioned above could be combined with the NTR-MTZ transgenic lines (**Table 1**), to target specific cell subtypes and elucidate the mechanisms that enable rewiring. Research in this front could have a very significant impact in phenotyping vision loss in RD, and to develop manipulations that could ultimately enable rewiring of transplanted or regenerated photoreceptors into proper retinal circuits.

### Gene-Expression Profiling (RNAseq)

During the last decade, advances in the capacity of high-throughput sequencing has allowed to profile the transcriptomes of whole tissues or dissociated single cells. Gene-expression profiling of retinal cells in mice has given great insight in the classification of retinal cells into different (and even novel) subtypes (Macosko et al., 2015), especially for BC, where clear differences in molecules involved in cell-recognition and synapse formation were detected (Shekhar et al., 2016). These techniques can be applied in zebrafish, especially using transgenic lines, as has been recently reported for rods (Sun et al., 2018). RNAseq of zebrafish retinal cells could help unveil the genes that are required for synapse formation between BC and photoreceptors, genes that could be essential to promote rewiring in RD therapies. RNAseq could also be exploited to study the changes in gene expression that occur during degeneration and regeneration. This could be accomplished by profiling single cells in the most relevant time points after photoreceptor death. With a focus on Müller glia, further insights could be gained into the gene networks that allow pluripotent and functional recovery in zebrafish. This knowledge will also be extremely valuable for the treatment of RD and understanding how to initiate regeneration after RD.

## Conclusion and Outlook

Our field is developing a deep understanding on many aspects of RD, including risk factors, underlying genetic causes, molecular pathways that lead to photoreceptor death, and the manipulations that could slow down the progression of the disease. During the last decade, we have made significant progress into revolutionary therapies that could, 1 day, cure blindness.

Despite all of the research on RD and regeneration, there are still gaps in our current knowledge that limit our capacity to understand certain aspects of RD and hinder our ability to develop therapies. The zebrafish is an advantageous model to fill in these gaps, especially at a mechanistic level. As a relevant example, we have discussed how zebrafish has been used to delineate molecular pathways within Müller glia that allow regeneration of retinal cells even in the adult zebrafish. This knowledge has been directly applied into the mouse retina and successfully used to generate new and functional bipolar and amacrine cells (Jorstad et al., 2017). Although studies have been able to stimulate retinal regeneration in mice using manipulations derived from the study of zebrafish retinal regeneration, but we do not yet fully understand the pathways required to regenerate each retinal cell type, or how these pathways are regulated to regenerate specific subpopulations. Further studies are required to understand the mechanisms that allow zebrafish Müller glia to not only produce any retinal cell, but also to specifically replace the lost population without overtly producing proliferation of undamaged cell types (D’Orazi et al., 2016; Yoshimatsu et al., 2016; McGinn et al., 2018).

In addition to gaps in knowledge on the role of Müller glia and regeneration, we also have an incomplete grasp on the processes that are involved in recognition between photoreceptors and their postsynaptic targets, with only a handful of molecules known to be involved in the correct formation of synapses. This leaves us with little leverage on manipulations that could promote integration of new photoreceptors into the surviving retinal circuits. Solving these issues and finding viable therapeutic options for RD will certainly require diverse approaches. Research in zebrafish is uniquely poised to make additional key contributions into RD, especially on unveiling the molecular mechanisms involved in photoreceptor regeneration and the processes that guide wiring during development and rewiring after regeneration of photoreceptors into retinal circuits.

## Author Contributions

JA conceived and carried out the literature review research, designed the figures and diagrams, acquired the images, and wrote the article. KK conceived and carried out the literature review research and wrote the article.

## Conflict of Interest Statement

The authors declare that the research was conducted in the absence of any commercial or financial relationships that could be construed as a potential conflict of interest.

## Footnotes

1 http://www.sph.uth.tmc.edu/retnet
